# Therapy of Skin, Hair and Nail Fungal Infections

**DOI:** 10.3390/jof4030099

**Published:** 2018-08-20

**Authors:** Roderick Hay

**Affiliations:** Kings College London, London SE5 9RS, UK; roderick.hay@ifd.org

**Keywords:** dermatophytes, cutaneous candidiasis, *Malassezia* infection, treatment, onychomycosis

## Abstract

Treatment of superficial fungal infections has come a long way. This has, in part, been through the development and evaluation of new drugs. However, utilising new strategies, such as identifying variation between different species in responsiveness, e.g., in tinea capitis, as well as seeking better ways of ensuring adequate concentrations of drug in the skin or nail, and combining different treatment methods, have played equally important roles in ensuring steady improvements in the results of treatment. Yet there are still areas where we look for improvement, such as better remission and cure rates in fungal nail disease, and the development of effective community treatment programmes to address endemic scalp ringworm.

## 1. Introduction

Fungal infections of the skin and its adnexal structures, such as hair and nails, are common in all regions of the world. Yet over the last 40 years, there have been huge advances in the management of these conditions, starting from a time when most of the available therapies were simple antiseptics with some antifungal activity, to the present day where there is a large and growing array of specific antifungal antimicrobials. Although the course to modern treatment has not been without its problems, the complications, commonly encountered amongst antibacterials, particularly drug resistance, have not had a major impact on the currently used antifungals, with the possible exception of the superficial *Candida* infections, where azole resistance is well recognised. This, combined with selection pressure, has led to an increase in organisms, such as *Candida glabrata*, with a lower level of azole sensitivity. The rise of *Candida auris* as a pathogen, which is frequently multidrug resistant, is a further worry, although to date, it has not had a major impact on skin infection, but superficial carriage is well documented. Drug toxicity is not a major problem, although, when encountered, even rarely, in the context of the management of non-life-threatening conditions, it presents a particular dilemma in risk management. The risk benefit ratio is different to that encountered with systemic mycoses; the withdrawal of recognition of oral ketoconazole for the treatment of superficial infections by regulatory authorities in Europe and the United States illustrates the point.

## 2. Fungal Infections of the Skin

### 2.1. Dermatophytosis

The results of medical treatment of most dermatophyte infections affecting the skin are now excellent, with cure rates ranging from 80–90%, and there is a correspondingly wide range of antifungal agents in use as both topical or oral formulations [1,2,3,4]. All these are effective in the majority of patients, providing they are used regularly and for the recommended durations. In most recent studies of antifungal efficacy for dermatophytosis, the primary endpoint for clinical trials is complete cure, which includes complete clinical cure [5] with mycological recovery as a secondary endpoint. This is not entirely satisfactory, as the timing of improvements in the clinically observable changes may lag behind the disappearance of the organisms themselves, in infections such as tinea pedis.

The treatment approach, usually adopted for dermatophytosis affecting the skin, is to use topical treatments for “localized” infections or those of limited spread and oral treatments for more extensive infections. Compliance with topical treatment is often an important issue, as many patients find repeated applications both time consuming and difficult [6]. For this reason, some antifungals have been assessed in trials as once-, rather than the more usual twice-daily treatment applications, or after a single or limited number of applications.

#### 2.1.1. Topical Treatments

A wide variety of topical applications has been used to treat dermatophyte skin infections [1,2,7,8,9,10]. Serious adverse effects are uncommon with this approach, and allergic or irritant contact dermatitis is also rare.

Imidazole preparations for topical use, such as clotrimazole, miconazole, econazole, and ketoconazole, are now well established as effective treatments in ringworm infections with a low incidence of adverse reactions [1,2,3]; other drugs in this group, such as tioconazole [11] and sulconazole [12], are equally effective. These older topicals have been joined by newer preparations such as sertaconazole [13], luliconazole [13], and isoconazole [14], although they have not been licensed in all countries. The azole antifungals are usually available in cream, solution, or spray formulations at a 1% concentration. Most are used twice daily for 2–4 weeks, although some, such as bifonazole, are licensed for once-daily use [15]. There is little difference in the efficacy of the different azoles [16].

An effective alternative treatment is the topical formulation of 1% terbinafine [17,18]. Terbinafine cream applied locally in dermatophytosis produces remissions in some dermatophyte infections, e.g., interdigital tinea pedis, after only short periods of application, e.g., 7 days. There is also a topical film forming solution of terbinafine, for infections of the foot and the sole, used as a single application [19]. The solution is applied and left to dry for 3–4 min. Other allylamines, such as naftifine [20] and butenafine [21], are also effective. Cyclopirox is available in some countries as a topical application for use in dermatophytosis [4].

Other older cream or powder preparations such as tolnaftate or zinc undecenoate can be purchased without prescription.

#### 2.1.2. Oral Antifungals

Oral antifungals are very useful dermatophyte infections of the skin (Table 1). Terbinafine is given orally in a dosage of 250 mg/day for dermatophytosis. It produces rapid and long-lasting remissions in dry type tinea pedis and tinea cruris, as well as tinea corporis after 2 weeks [22]. A smaller tablet form of 125 mg is available in some countries for treatment of children. Itraconazole is active against a wide range of dermatophytes, and is effective in regimens of 100 mg for 2 weeks in tinea cruris and corporis, or 30 days in dry type tinea pedis [23]. The currently preferred regimen uses 400 mg/day for one week in tinea corporis, and two weeks in dry type tinea pedis [24,25]. Occasionally, longer periods of treatment are needed. There is a new and more regularly absorbed formulation of itraconazole, which is available in some countries [26]. Fluconazole is given as a daily treatment of 50 mg for 2–4 weeks in dermatophyte infections of the skin [27]. It can also be used in a regimen of 150 mg/week for 2–3 weeks for tinea corporis and tinea cruris, but longer for dry-type tinea pedis [28,29].

Ketoconazole is an alternative agent given in a 200–400 mg/day regimen for adults. Hepatitis is a known complication, occurring at a frequency of approximately 2.9 events/1000 person-years [30] and because of this, ketoconazole is no longer used in Europe and the United States for superficial infections. For tinea infections there is little data, at present, on the use of posaconazole [31] and voriconazole [32], apart from unusual cases of deep dermatophytosis. Although newer oral drugs, such as pramiconazole in tinea corporis [33], ravuconazole in onychomycosis [34], and albaconazole [35] have been tested in vitro or by phase 1 to 2 clinical trials in dermatophyte infections, at present, they are not marketed for these indications. Griseofulvin is largely restricted to use in dermatophyte infections at a dose of 10 mg/kg/day given in tablet form [25]. There is a solution form for children, although this is no longer available in many countries. Treatment duration varies between 2 and 4 weeks for tinea corporis or cruris.

Some tinea infections, usually caused by *T. rubrum*, at sites such as the groin or the trunk which, while responding initially to treatment with either terbinafine or itraconazole, relapse quickly [36]. Different treatment regimens have been tried anecdotally, including combinations of azole or allylamine oral medications, plus topical azoles or allylamines. At present, there is no consistently effective remedy in these cases. There has been a similar problem reported from India where persistent tinea corporis or cruris is associated with extensive lesions [37]; many of these cases are caused by *T. interdigitale.* By contrast, tinea infections of the skin in immunosuppressed patients, including those with HIV/AIDS, usually respond to treatment, although it is often necessary to double the normal dose [38].

### 2.2. Candida Infection of the Skin

*Candida* infections of the skin respond well to a range of antifungals available in cream, powder, or solution formulations [39,40]. Useful antifungals for candidosis are azole drugs (econazole, clotrimazole, ketoconazole, and miconazole). Other antifungal agents, not suitable for dermatophytosis, are the topical forms of the polyene antifungal drugs, such as nystatin, amphotericin B, and natamycin [41]. Generally, superficial infections due to *Candida* of the skin and mucous membranes respond well to these treatments. However, most clinical trials have focused on oropharyngeal or vaginal infections, or cutaneous involvement in chronic mucocutaneous candidiasis (CMC).

The most commonly used oral treatments for candidosis are fluconazole [42] and itraconazole [43], most studies focusing on the mucosal infection or CMC. The usual daily doses are itraconazole 100–200 mg, and fluconazole 100–400 mg. Resistance to fluconazole is an established issue which has been reported in HIV/AIDS or CMC patients receiving long-term therapy. Within an infected area such as the mouth, there may be both sensitive as well as resistant strains of *Candia* isolated in oral infection, showing that there is heterogeneity of the population in an infection [44]; it is not clear if this also applies to the skin. Characteristically, patients at risk from drug resistance are those with persistent infection requiring long-term suppressive treatment and who are immunosuppressed, and usually, the infection is oral or intravaginal, rather than cutaneous. Primary drug resistance to fluconazole has been recorded with some *C. albicans* species, and with *C. krusei*, *C. dubliniensis*, *C. glabrata*, and *C auris*. However, *Candida* resistance is less common in patients under treatment for candidosis who are receiving highly active antiretroviral (HAART) therapy. Other azoles active against *Candida* species include voriconazole and posaconazole [45,46]. Both of these have been used for severe oropharyngeal and oesophageal infection in the seriously ill, but not *Candida* skin infections.

Flexural candidosis. This refers to *Candida* infection in flexures such as the groins or inframammary fold, sometimes called *Candida* intertrigo. This requires topical therapy (azole or polyene creams) given for 2 weeks [1,2], but treatment may be continued for longer periods. Drying the infected site is also important in many cases where the moist skin surface can cause much discomfort.

### 2.3. Malassezia Infections

Pityriasis versicolor. A wide range of different antifungal drugs is effective in pityriasis versicolor [47], and cure rates of over 85% can be achieved. Topically applied azole antifungals, such as miconazole, clotrimazole, ketoconazole, and sertaconazole work well in pityriasis versicolor, and there is no difference in results achieved by different antifungal compounds [48]; most commonly, azole creams are used. Topically applied allylamines, such as terbinafine 1% cream, naftifine, or butenafine [49], as well as ciclopirox, are also effective in pityriasis versicolor. The usual time to recovery with all treatments is 2–3 weeks. One of the practical drawbacks in treatment of this condition is the wide surface area involved. One approach to circumvent this is to use a medication which can be spread more widely over the body e.g., as a mousse, like ketomousse [50]. Ketoconazole shampoo has not been formally evaluated in pityriasis versicolor, but two or three applications of the shampoo over a week appear to clear most infections. A second approach is the application of 2.5% selenium sulphide in a detergent base (Selsun^®^ shampoo) [51]. It is applied to all the affected areas and left overnight. In many cases, it is necessary to apply the material regularly, e.g., every other night over 2 weeks.

Oral itraconazole is also very effective in cases of pityriasis versicolor, although it is mainly used for extensive or recalcitrant cases [52]. Itraconazole is active against pityriasis versicolor in a total dosage of 800–1000 mg, usually given over 5 days. Fluconazole is an alternative [53]. 

The best treatment for *Malassezia* folliculitis is oral. For instance, itraconazole is given orally at a dose of 100 mg daily for 2–3 weeks [54]. This infection responds less consistently to topical antifungal treatment probably because of the need to ensure good penetration into the hair follicles.

In seborrhoeic dermatitis, there have been a few studies of the efficacy of azole antifungals. In a systematic review [55] on the subject, there was evidence that medications containing ketoconazole applied topically either to the scalp or facial skin were most effective. Likewise, there are also studies with bifonazole and selenium sulphide that demonstrate efficacy. In practice, other azoles appear to be effective and, in extensive cases, oral itraconazole 100 mg daily can be usually used for 10–14 days to induce a remission. The practical problem with this condition, and whatever treatment is used, is that it relapses often, and on a regular basis, and patients should be made aware of this.

### 2.4. Tinea Capitis

Topical antifungal therapies have little place in the management of tinea capitis, except as adjuncts to oral therapy to limit spread or treat carriers [56]. Treatment for tinea capitis, which is largely confined to children, involves the use of oral antifungals (Table 1), such as terbinafine, itraconazole, griseofulvin, or fluconazole [57,58,59,60]. There is no clinical evidence to support the use of other oral antifungals, including the newer azoles, such a voriconazole or posaconazole. Single-dose therapy with griseofulvin and intermittent dose regimens (25 mg/kg twice a week) have had some success, but in general, conventional daily therapy is advisable (10–15 mg/kg), and treatment durations of at least 6 weeks are usually adequate. It is used particularly in cases caused by *Microsporum* species. In some infections, such as those caused by *T. tonsurans*, much longer courses and sometimes higher dosage (20 mg/kg/day) of griseofulvin therapy may be needed [56]. A suitable liquid formulation of griseofulvin is not available in all countries.

Terbinafine, although not licensed in all countries for use in children, is the preferred alternative for certain infections, such as those caused by *Trichophyton* species, although there are fewer data, and the drug appears to be less effective in disease caused by *Microsporum* species [58,59]. The best length of treatment for *T. tonsurans* and *T. violaceum* infections with terbinafine appears to be 1 month. There is some evidence that higher doses (double the standard dose) of terbinafine may be more effective for *Microsporum*. The appropriate length of treatment with either itraconazole or fluconazole is not established, although both appear to be effective against *T. tonsurans*. 

Dosing regimens for tinea capitis in children are shown below:

Terbinafine <10 kg 62.5 mg, 10–20 kg 125 mg, >20 kg 250 mg—all daily for 4 weeks
Itraconazole 2–4 mg/kg/day for 4–6 weeks
Griseofulvin 10 mg/kg 6–8 weeks (20mg/kg considered in some *T. tonsurans* infections)

A systematic review, using additional published data, suggested that for *Trichophyton* infections of the scalp, terbinafine was preferable, whereas griseofulvin was favoured in *Microsporum* infection [59].

Ketoconazole shampoo or selenium sulphide is used 2–3 times weekly to prevent spread from patients in the early phases of therapy, in combination with an oral treatment. A similar approach is used for siblings of patients with anthropophilic tinea capitis. If scalp scrapings or brushings are positive on culture, but the scalp is clinically normal, they are thought to be carriers, whose scalp contains viable fungal organisms, but without hair shaft invasion [57,60,61], and many advise treating with antifungal shampoos, as described above.

### 2.5. Onychomycosis

In fungal nail infections, there have been a number of new developments designed to provide better cure rates, either by improving the capacity for nail plate penetration by antifungals, or through combining antifungal therapy with removal of the infected nail plate, either through surgical excision or laser ablation; other new approaches include the use of photodynamic therapy and iontophoresis. As with other superficial mycoses, most recent clinical trials use complete cure as the target endpoint. This is defined as clinically normal nail combined with mycological cure. Older studies used different endpoints, for instance through including, as cures, nails with minimal residual clinical change. This has made comparison between different studies difficult. A further problem in interpretation has been variation in study and follow-up durations. With many of the newer combined approaches to treatment there have been few well-documented clinical trials with adequate follow-up periods.

#### 2.5.1. Onychomycosis Due to Dermatophytes

##### Topical Therapies

Recently, topical therapy for onychomycosis has been made possible by the development of several novel antifungal drug formulations designed to improve nail penetration. Topical therapy has the perceived advantage of safety, without laboratory monitoring. Compliance with a regular topical regimen is often poor though, with prolonged regimens given over several months. Generally, topicals are only used where there is no nail matrix involvement or significant nail plate thickening.

Azole antifungal agents. The 1% cream or solution formulations of the older topical azole antifungals, such as clotrimazole and miconazole, have been used in onychomycosis without striking success. However, several novel azoles have also been developed to address nail treatment specifically. The first of these to be introduced, tioconazole and bifonazole, are two such azoles that have been used topically in the treatment of onychomycosis. Tioconazole is formulated as a 28% solution. It has been found to produce a clinical cure at 3 months after therapy in onychomycosis of the fingernails and toenails, at 22% [62]. Bifonazole, another topical imidazole, has been used in a 40% urea paste formulation. Urea containing bifonazole is applied to the nail plate under occlusion until the nail softens and is easily debrided, and patients then apply bifonazole cream daily as the nail grows out [63]. The newest topical treatments have involved the use of new azole agents. Efinaconazole is a triazole antifungal applied once daily, and is applied directly to the nail plate and surrounding skin. It has demonstrated low affinity for keratin, which enhances nail penetration, Efinaconazole is available in some countries as a 10% solution. Complete cure rates range from 15–18% [64,65]. Luliconazole, another new azole in a 5% solution with similar efficacy rates, is a broad-spectrum imidazole. The solution formulation demonstrated a favourable safety and tolerability profile [66]. These preparations are available in some countries for the treatment of onychomycosis.

Amorolfine is available in a transungual delivery system. After application of the 5% lacquer, the solvent evaporates in 3–5 min, increasing the concentration of amorolfine in the film to 27% at the nail surface. Complete cure was reported in 46% and 52% of patients receiving once- and twice-weekly treatment, respectively. However, it is important to note that complete cure for this study was defined as negative mycology and <10% nail involvement [67]. This is difficult to compare with more recent studies with newer drugs using more stringent criteria.

Ciclopirox is a hydroxypyridone derivative that acts by chelation of trivalent cations, such as Al^3+^ and Fe^3+^, resulting in inhibition of metal-dependent enzymes, which disrupts the transport of nutrients and amino acids. It is available as an 8% lacquer for the treatment of onychomycosis. In two studies, complete cure rates, using stringent criteria of 5.5–8.5%, were found [68,69] 

Tavaborole is another new antifungal agent available as a 5% solution. It is a member of a new class of boron-containing antifungals that inhibit fungal protein synthesis by targeting fungal cytoplasmic leucyl-transfer ribonucleic acid (tRNA) synthetase. In two double-blind vehicle-controlled studies in which 5% tavaborole solution was applied once daily for 48 weeks, complete cure rates of 6.5% and 9.1% were recorded [70].

There are other topical antifungals for use in nail disease in development, including those utilizing terbinafine as the active compound. Generally, data on long-term relapse rates with the new topicals is still sparse, although this may change with longer experience.

##### Oral Antifungals

Oral antifungal agents have achieved greater success rates than topical therapies for onychomycosis (Table 1). Terbinafine is one of the most commonly used antifungals to treat dermatophyte onychomycosis, as well as other dermatophyte infections. The dose in onychomycosis was 250 mg daily for 12 weeks for toenails, and 6 weeks for fingernails. There are many studies with slightly differing assessment criteria which have reported complete and mycological cure rates of 38% and 70% [71,72,73]. There are also some comparative studies. For instance, oral terbinafine at 250 mg daily for 12–16 weeks demonstrated superior efficacy compared to the older agents itraconazole, fluconazole, and griseofulvin [54]. In a trial comparing continuous terbinafine therapy to intermittent itraconazole therapy, terbinafine produced a complete cure rate of 35% (vs 14%) [74]. Caution should be used in patients with liver dysfunction. Many physicians periodically monitor liver enzymes, but recent data suggest that this is not always necessary.

Itraconazole is given at a dose of 200 mg daily for 6 weeks for fingernail onychomycosis and 12 weeks for toenail onychomycosis or, as a pulsed regimen, intermittently as 400 mg daily for one week per month for 3–4 consecutive months for toenail infections, and 2 months for fingernail infections [75]. In a meta-analysis of randomized controlled trials, mycological cure rates of itraconazole averaged 59% for continuous therapy and 63% for pulsed therapy [76]. Another meta-analysis of studies, comparing continuous and pulsed regimens, showed average clinical and mycological cure rates for toenail onychomycosis at 12 months follow-up, after the start of therapy, of 86% and 74%, for continuously dosed itraconazole at 200 mg daily for 3 months. For toenail onychomycosis treated with three pulses of itraconazole at 400 mg daily for one week per month, the average clinical and mycological cure rates were 82% and 77% at 12 month follow-up. The cure rates were higher for fingernail onychomycosis [77].

Side effects of itraconazole include gastrointestinal and neurological side effects (headaches, dizziness), and more rarely, hepatotoxicity and morbilliform or pustular skin rashes can occur. A serious but very rare side effect is congestive heart failure. As with oral terbinafine, laboratory monitoring may be required in some patients. 

Fluconazole has also shown efficacy in onychomycosis [78]. The usual dosage of fluconazole for onychomycosis is 150 mg once weekly for 6 months for fingernails, and 12 months for toenails [79]. Clinical cure (completely healthy-appearing nail) rates at the end of treatment averaged between 28–36%.

Posaconazole, given at a dose of 200 mg daily as an oral suspension for 24 weeks, is the most effective dosing regimen with this drug for obtaining a complete cure (54.1%). Hence, posaconazole may provide an alternative for patients unable to tolerate terbinafine, or those with nondermatophyte infections [80], however, at present, it is expensive.

##### Other Treatments for Onychomycosis

Medical (non-pharmaceutical) devices or approaches are increasingly important options in the treatment of onychomycosis, either used on their own or in combination with other topical or oral antifungal treatments. The techniques that have currently been tested include ultraviolet radiation, iontophoresis, and lasers; however, at present, there are no randomized controlled trials evaluating devices and/or combination treatments, and no long-term follow-up data [81,82,83].

Laser therapy is based on the principle of selective photothermolysis. This involves targeting specific chromophores causing local destruction with minimal damage to the surrounding healthy tissue. Many studies have investigated the use of lasers in the treatment of onychomycosis. A review of 22 studies showed that lasers are generally well-tolerated with only mild adverse effects. However, the variability in the design of individual studies makes drawing valid conclusions difficult. These studies have usually recruited small patient samples, and there has also been variation in treatment regimens and endpoints. As described previously, the cure criteria were inconsistently defined. Currently, there is no conclusive evidence that laser therapy is effective in the long term for onychomycosis, although immediate results in terms of appearance are usually good [84,85].

Nd:YAG laser systems are the most common laser devices used for the treatment of onychomycosis. Diode lasers operate at temperatures that are safe for human tissues, and ablative lasers include carbon dioxide and erbium lasers, and have also been used [67]. Combining laser treatment with a specific antifungal treatment, such as topical amorolfine, may also prove an effective strategy [86,87], but more data are needed.

Photodynamic therapy (PDT) utilizes a phototoxic reaction that occurs following topical application of photosensitizing agents, such as 5-aminolevulinic acid (ALA), methyl aminolevulinate, and methylene blue (MB), and exposure to a light source. It is widely used in the treatment of some skin cancers. Again, evaluation of its use in onychomycosis, although promising, is hampered by a lack of stringent controlled trials with adequate follow-up data [88,89,90].

Other physical methods include iontophoresis, which involves transport of pharmacologically active molecules into the nail by applying a low voltage electric field, and has been utilised for drugs such as in a gel formulation of terbinafine. Surgical treatments can be divided into simple mechanical debridement or chemical (40% urea) or surgical nail plate avulsion [91,92,93,94]. Again, these can be combined with either oral or topically administered antifungals. A further technique has been the use of multiple drilled sites to create small wells in the nail plate, followed by application of different antifungals, such as butenafine or terbinafine, into the holes created in the nail plate surface, with subsequent diffusion of the active compounds into the nail tissue [95].

#### 2.5.2. Onychomycosis Caused by Other Organisms

Nail dystrophy caused by *Candida* can be treated with oral itraconazole, fluconazole, or ketoconazole, or chemical removal followed by local antifungal treatment [96]. If these methods are unsuccessful, combined avulsion and antifungal therapy should be used. In chronic mucocutaneous candidosis, the daily dose of itraconazole may have to be increased to 200 mg daily. When remission is induced, treatment should be stopped. Resistance to this drug has only rarely been recorded. With *Candida* paronychia the infection by *Candida* is often compounded by bacterial invasion of the nail fold and secondary irritant or contact dermatitis. In this situation, the use of oral or topical antifungals is often combined with topical corticosteroids. The condition frequently relapses because the swollen nail fold remains a potential portal of entry for both microbes, as well as irritants. This can be helped by a surgical technique that removes the fibrotic tissue that is found in chronic nail fold swelling [97].

Treatment of other fungal infections of the nail, such as those caused by *Fusarium*, *Neoscytalidium*, or *Scopulariopsis*, is largely based on anecdotal experience, as case numbers are too few to set up a clinical trial. Often in these cases, a combination of therapeutic approaches, including oral and topical, e.g., amorolfine, antifungals, and nail plate ablation, are used.

## 3. Summary

There have been huge advances in the management of most fungal infections of the skin that have encompassed new antifungal drugs, new formulations, as well as ancillary physical approaches to therapy. The results are impressive. There are still some areas where there is work to be done. The treatment of many fungal infections affecting the nails still remains lengthy, and is often accompanied by uncertain or even poor responses. Shorter, efficacious treatments for onychomycosis, backed by convincing clinical trial evaluations, are needed. There is also a problem with the treatment of many superficial fungal disease in resource-poor settings where, for instance, in tinea capitis, prevalence rates can exceed 25% in school age children. Affordable treatments in these situations, backed by a public health strategy, would be the route to successful control. While drug resistance in superficial fungal infections is not seen to be a significant problem, the continuing outbreak of recalcitrant and treatment-unresponsive dermatophyte infections, described previously in India, is of concern [37]. Although there is no evidence to suggest an immediate risk of spread, sporadic imported cases are being recognized increasingly in other countries. Learning more about the reasons for treatment failure in normally responsive infections is a priority.

## Figures and Tables

**Table 1:** Systemic treatment for dermatophyte infections (summary).

	First line	Alternative
Tinea pedis (dry type)	**Terbinafine** 250 mg/day for 2 weeks **Itraconazole** 200–400 mg/day for 1 week	**Fluconazole** 6 mg/kg/week for 4–6 weeks
Tinea corporis (extensive)	**Terbinafine** 250 mg/day for 1 week **Itraconazole** 200 mg/day for 1 week	**Fluconazole** 150–200 mg/week for 2–4 weeks
Onychomycosis due to dermatophytes *	**Terbinafine** 250 mg/day for 12 weeks (toe nails) or 6 weeks (fingernails) **Itraconazole** 200 mg bid for 1 week/month for 3 months (toe nails) or 2 months (finger nails)	**Fluconazole** 150–200 mg/week for 6–9 months (toe nails) or 3–4 months (finger nails)
Tinea capitis (children)	**Terbinafine** 125 mg (<25 kg), 187.5 mg (25–35 kg) or 250 mg (>35 kg) daily for 3–4 weeks Mainly *Trichophyton* infections **Griseofulvin** 10–15 mg/kg/day for 6–8 weeks Mainly *Microsporum* infections	**Itraconazole** 5 mg/kg/day (maximum 500 mg) × 4–8 weeks

* In extensive infections, including those involving the nail matrix combination with an oral and topical antifungal, e.g., amorolfine or ciclopirox, is useful. This may have to be combined with surgical removal e.g., after 40% urea or laser ablation—but there are few clinical trials.
